# Postprandial Glucagon Action in the Human Brain

**DOI:** 10.1111/dom.70801

**Published:** 2026-04-22

**Authors:** Robert Wagner, Stephanie Kullmann, Julia Hummel, Katsiaryna Prystupa, Elena Hosenfeld, Ralf Veit, Andreas L. Birkenfeld, Hans‐Ulrich Häring, Hubert Preissl, Andreas Fritsche, Andreas Peter, Martin Heni

**Affiliations:** ^1^ Institute for Clinical Diabetology, German Diabetes Center, Leibnitz Center for Diabetes Research at Heinrich Heine University Düsseldorf Düsseldorf Germany; ^2^ German Center for Diabetes Research (DZD e.V.) Neuherberg Germany; ^3^ Department of Endocrinology and Diabetology Medical Faculty and University Hospital Düsseldorf, Heinrich Heine University Düsseldorf Düsseldorf Germany; ^4^ Institute for Diabetes Research and Metabolic Diseases of the Helmholtz Center Munich at the University of Tübingen Tübingen Germany; ^5^ Department of Internal Medicine IV, Division of Diabetology, Endocrinology and Nephrology Eberhard Karls University Tübingen Tübingen Germany; ^6^ Division of Endocrinology and Diabetology, Department of Internal Medicine I University of Ulm Ulm Germany; ^7^ Institute for Clinical Chemistry and Pathobiochemistry, Department for Diagnostic Laboratory Medicine University Hospital Tübingen Tübingen Germany

**Keywords:** brain metabolism, cerebral blood flow, glucagon, hypothalamus, insulin sensitivity, metabolic regulation, MRI neuroimaging, OGTT (oral glucose tolerance test)

## Abstract

**Aims:**

Elevated fasting glucagon is linked to hyperglycemia, but postprandial glucagon effects are less understood. Recent evidence suggests metabolic benefits of rising glucagon after oral glucose intake, potentially impacting brain‐mediated whole‐body metabolism. To elucidate the translational relevance of these findings, we studied postprandial effects of glucagon on the human brain.

**Materials and Methods:**

We performed oral glucose tolerance tests (OGTT) combined with functional magnetic resonance imaging to quantify brain activity and connectivity at fasting, 30 and 120 min post glucose load in 30 volunteers. In 14 participants with suppressed glucagon, low‐dose glucagon infusion mimicked non‐suppressed glucagon after OGTT. This was compared to 7 participants with endogenous rising glucagon during OGTT.

**Results:**

Low‐dose glucagon infusion did not elevate plasma glucose levels during OGTT. Also, no changes in insulin sensitivity and insulin secretion were observed. However, experimentally elevating glucagon during OGTT in individuals with physiological suppression of glucagon significantly increased postprandial brain responsivity in the hippocampal gyrus and in brain regions important for the homeostatic and hedonic regulation of food intake as well as systemic metabolism (i.e., hypothalamus and ventral striatum). Most postprandial brain responsiveness during glucagon infusion was directionally consistent with the findings in persons with endogenously rising glucagon. Moreover, the postprandial brain response correlated with the rise in glucagon, regardless of exogenous or endogenous source of glucagon. Although the overall glucagon trajectory during OGTT was not significantly different over the full 0–150 min period, the groups differed at key post‐challenge timepoints and in integrated glucagon exposure. Together with the infusion and correlation analyses, this supports a relationship between postprandial glucagon and brain responsivity, while more subtle differences in glucagon kinetics will require larger studies.

**Conclusions:**

Our findings demonstrate postprandial effects of glucagon in metabolically relevant human brain areas. This may underlie the promising effects on body weight achieved with pharmacological multi‐agonists that activate the glucagon receptor.

## 1 Introduction

The peptide hormone glucagon is produced in pancreatic alpha cells and transported to distant organs through the bloodstream. It has long been thought to be the counterplayer to insulin as its pharmacological administration transiently raises blood glucose and elevated fasting concentrations of the hormone are present in patients with type 2 diabetes [1]. Indeed, elevated fasting glucagon is associated with insulin resistance, which is a hallmark of type 2 diabetes. However, recent research suggests a more complex role of glucagon in human metabolism. Glucagon stimulates beta‐oxidation of fatty acids [2] as well as hepatic lipolysis and inhibits the release of very‐low‐density lipoprotein (VLDL)‐associated triglycerides from the liver [3, 4]. It also regulates hepatic amino acid turnover and circulating amino acid levels [5].

Based on data from three independent cohorts, we reported that stable or even rising glucagon concentrations from fasting to 120 min during an oral glucose intake are linked to lower prediabetes risk, higher insulin sensitivity, and lower liver fat [6]. In agreement with these findings, administration of the glucagon receptor antagonist LY2409021 led to higher post‐challenge glucose in a clinical study [7]. Glucagon also increases energy expenditure, lowers appetite, and reduces body weight in humans [8].

After oral glucose intake, glucagon is typically suppressed by the combined action of rising glucose, islet‐ and incretin‐mediated feedback, and likely also by feedback within the alpha‐cell–liver axis, in which hepatic amino acid handling contributes to the regulation of alpha‐cell glucagon secretion [5]. However, a subset of individuals show stable or rising glucagon concentrations after oral glucose challenge. In our previous work, this non‐suppression phenotype was associated with higher insulin sensitivity, lower liver fat, and lower prediabetes risk, suggesting that it may reflect a metabolically favourable state rather than dysregulation [6]. As glucagon also influences appetite and body weight, functions that are strongly regulated by the brain, we hypothesised that central nervous system mechanisms may contribute to these associations. We therefore investigated postprandial glucagon action in the human brain and tested whether low‐dose glucagon infusion can mimic the brain response seen in individuals with endogenous non‐suppressed glucagon.

Glucagon is detectable in human cerebrospinal fluid [9, 10] and glucagon receptors have been identified in the central nervous system [11]. Recent work further demonstrated glucagon receptor expression in human brain tissue beyond the blood–brain barrier, particularly in neurons of the frontal cortex, with variable expression across brain regions [12]. Injection of glucagon directly into the brain reduced food intake in sheep [13]. In addition to possible direct glucagon signalling in brain cells [14], the brain is likely further informed on portal vein glucagon concentrations via neuronal signals from the periphery to the brain [1]. Besides the established glucose regulating properties of glucagon action in the liver, glucagon injection directly into the brain modulates whole‐body glucose and lipid metabolism in different species (reviewed e.g., in [10]).

Recent research indicates that the human brain has the ability to profoundly modulate whole‐body homeostasis [15, 16] and alters long‐term body weight and body fat distribution trajectories [17]. It is therefore tempting to speculate that the brain action of glucagon can also contribute to metabolic effects in the entire organism. This could be of special importance in the postprandial state, when modulatory outflows from the brain to the periphery appear to have the strongest contribution to the regulation of whole‐body metabolism [17]. Brain glucagon action could thus contribute to the healthier metabolic phenotype observed in persons with non‐suppressed glucagon after oral glucose intake, and play a role in the favourable pharmacologic effects of glucagon‐agonists currently being tested in advanced phases of clinical studies in combination with Glucagon‐like peptide 1 (GLP‐1) and/or Gastric inhibitory peptide (GIP) agonists [18, 19]. However, if, where, and to what extent glucagon acts in the human brain under physiological postprandial conditions remain unclear. We therefore studied brain effects of physiologic glucagon concentrations during an oral glucose tolerance test [20].

## 2 Materials and Methods

### 2.1 Participants and Intervention

**FIGURE 1:**
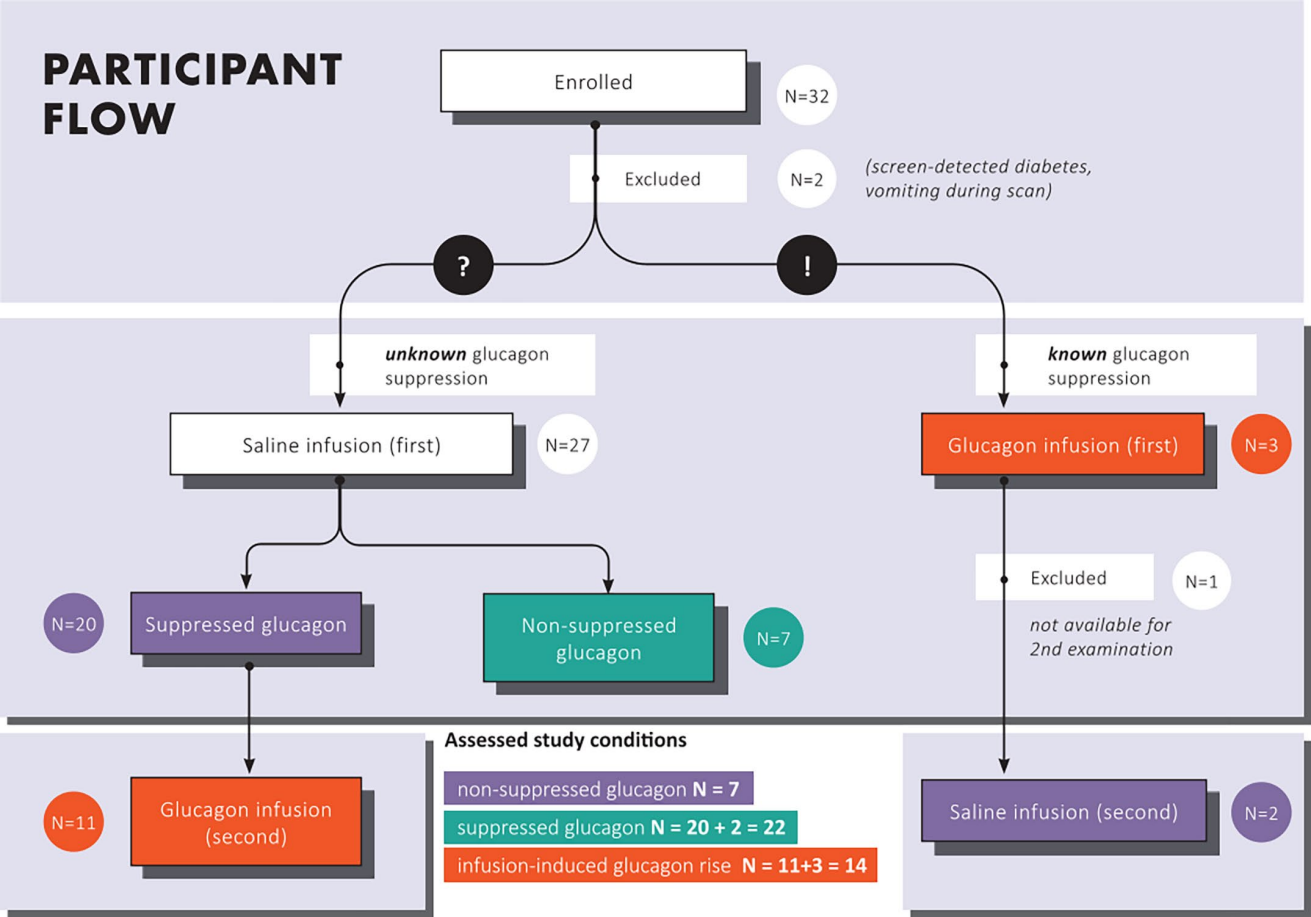
Study design.

The study was conducted at the University of Tübingen, Germany between February 2017 and January 2019. We initially enrolled 32 healthy adults. Three of them were previously characterised as participants with suppressed glucagon [21], and were pseudo‐randomised to a first intervention with glucagon infusion. The glucagon dose was chosen to mimic the physiological post‐challenge glucagon rise observed in our earlier work. For dose selection, we used the human infusion study by Cegla et al. [22] as an anchor, in which glucagon was infused at 2.8 pmol/kg/min and produced a substantially larger plasma glucagon rise than the endogenous increase we aimed to reproduce. We extrapolated that about 0.5 pmol/kg/min for the dose mimicking non‐suppressed glucagon. From the other study arm with saline infusion first, one participant was excluded because the 75 g oral glucose tolerance test (OGTT) revealed a previously unknown diabetes mellitus, and one participant was excluded because of vomiting during the MRI scan and OGTT; thus, 30 individuals were included in the study (Figure 1). Characteristics of the participants at first measurement are reported in Table 1. One participant who received glucagon infusion did not increase glucagon levels from fasting to 120 min but was kept in the intention‐to‐treat analyses. Repeated OGTT's were performed in a subset of participants with after a lag of 198 ± 82 days. All participants were invited to undergo a second measurement, and participation was based on availability and willingness to return for an additional study visit, resulting in 13 participants completing the crossover component of the study (Figure 1). All participants provided informed written consent. The study was pre‐registered at clinicaltrials.gov (NCT03061227). The local ethics committee approved the study protocol.

**TABLE 1:** Anthropometric and metabolic characteristics of the participants at the first measurement shown as descriptive statistics (mean and SD or *n* (%)).

Measure	Overall
*N*	30
Females/males	14/16
Age (years)	43 (15)
BMI (kg/m2)	25.9 (4.6)
HbA1c (%)	5.47 (0.39)
Fasting Glucose (mmol/l)	5.2 (0.5)
Glucose at 30 min during OGTT (mmol/l)	8.0 (1.2)
Glucose at 120 min during OGTT(mmol/l)	5.5 (1.7)
AUC(0–120) glucose (mmol/l*120 min)	834 (186)
Fasting Glucagon (pg/dl)	76.6 (43.7)
Glucagon at 30 min during OGTT (pg/dl)	72.4 (43.2)
Glucagon at 120 min during OGTT (pg/dl)	72.8 (49.7)
AUC(0–120) glucagon (pg/dl*120 min)	9128 (5724)
HOMA‐IR	2.0 (1.3)
Insulin sensitivity, OGTT‐derived (arbitrary units)	14.3 (7.5)
Insulin secretion (arbitrary units)	281 (88)

### 2.2 Measurements

All measurement time‐points and assessed analytes during OGTT are shown in Table S1. Plasma glucose and non‐esterified fatty acids were measured using the ADVIA Chemistry XPT autoanalyser (Siemens Healthineers) in sodium‐fluoride plasma. Serum insulin and C‐peptide levels were determined using the ADVIA Centaur XPT immunoassay system (Siemens Healthineers). Plasma amino acids were quantified using the Biocrates AbsoluteIDQ p180 targeted metabolomics kit (Biocrates Life Science AG, Innsbruck, Austria).

To measure total GIP, total GLP‐1, and glucagon, EDTA plasma samples collected at predefined OGTT timepoints (0, 30 and 120 min; see Table S1 for full sampling scheme) were treated with 300 ng/mL of aprotinin (Sigma, Merck, Germany). These samples were then processed at 4°C and stored at −80°C until analysed collectively. Plasma glucagon was measured in all participants with a commercially available radioimmunoassay (Millipore, St. Charles, MO). In a subgroup of 27 participants, GLP‐1 and GIP were additionally measured with commercially available ELISA assays (Mercodia, Sweden).

The Matsuda index [23] was calculated as 10 000 / square root of [fasting glucose × fasting insulin × mean glucose during OGTT × mean insulin during OGTT], using glucose and insulin values at 0, 15, 30, 60, 90 and 120 min. This index provides an OGTT‐derived estimate of whole‐body insulin sensitivity. Insulin secretion was estimated as area under the curve (AUC) C‐peptide(0–30) / AUC glucose(0–30), reflecting early post‐challenge beta‐cell response relative to glycemia [24]. AUC was calculated with the trapezoid method using all available measurement timepoints. Differential AUC of non‐esterified fatty acids (NEFA), to express suppression of NEFA, was calculated as (baseline NEFA level × 120) ‐ AUC NEFA(0–120), with higher values indicating stronger suppression of circulating NEFA during the OGTT. Glucagon suppression was defined as a lower glucagon concentration at 120 min than at fasting.

### 2.3 Brain Imaging Procedures

Scanning was conducted at a 3 T whole‐body Siemens scanner (Magnetom Prisma; Erlangen, Germany) with a 20‐channel head coil. Two types of functional datasets were recorded at each visit before, 30 and 120 min after oral glucose ingestion, each with an approximate acquisition time of 10 min. In addition, a high‐resolution T1‐weighted anatomical image was acquired once at baseline.

To acquire cerebral blood flow (CBF) maps, pseudocontinuous arterial spin labeling (pCASL) based on an echo planar imaging (EPI) sequence with background suppression was obtained with the following parameters: TR: 4 s, slice thickness: 4.5 mm, gap: 0.9 mm, TE: 14 ms, 16 slices, matrix: 64 × 64, bandwidth: 2004 px/Hz, tag gradient strength: 7.0 mT/m, bolus duration: 1.8 s [25, 26]. The imaging series consisted of 53 images whereby the first image of the series (M0) was used for calibration.

To assess resting‐state functional connectivity, whole‐brain blood‐oxygen‐level‐dependent (BOLD) data were collected by using multi‐band accelerated echo‐planar imaging sequences, developed at Center for Magnetic Resonance Research (CMRR) Minnesota, USA; with the following sequence parameters: TR = 1.5 s, TE = 34 ms, FOV = 192 mm^2^, matrix 96 × 96, partial Fourier = 6/8, bandwidth = 2264 Hz/pixel, echo spacing = 0.55 ms, flip angle 70°, voxel size 2 × 2 × 2 mm^3^, slice thickness 2 mm. Images were acquired in interleaved order with a multiband acceleration factor of 3. Each brain volume comprised 72 axial slices and each functional run contained 220 image volumes.

### 2.4 Image Processing

#### 2.4.1 Cerebral Blood Flow

Imaging preprocessing was performed using FSL (FMRIB Software Library version 5.0.9) and SPM (Statistical Parametric mapping, SPM12). After motion correction and realignment using mcflirt the ASL data was analysed using oxford_asl as part of the BASIL toolbox in FSL [27]. Perfusion images were obtained by pairwise subtracting of label and control images. A single‐compartment kinetic model with default parameter values and slice timing correction was used. Voxelwise calibration was performed as suggested by the white paper [28]. Thereafter, the M0 image was used for coregistration with the structural T1 image. The resulting transformation parameters were applied to the individual CBF maps. Subsequently the CBF images were normalised to an isotrop voxel size of 3mm [3] and smoothed with a 3D Gaussian kernel of 6 mm using SPM12.

#### 2.4.2 Resting‐State Functional Magnetic Resonance Imaging (fMRI)

We used the Data Processing Assistant for Resting‐State fMRI (67) to analyse the resting state fMRI data, which is based on SPM12 and Resting‐State fMRI Data Analysis Toolkit. The main steps are as follows: (1) Slice timing; (2) Head movement correction; (3) Registration of the structural image to functional image; (4) Spatial segmentation using 3D T1‐weighted images; (5) Functional image data normalised to the Montreal Institute of Neuroscience (MNI) space, and re‐sampled to 2 × 2 × 2 mm^3^ pixel size; (6) Nuisance variables removed (including white matter, CSF signal and 24 head parameters); (7) Bandpass filtering (0.01–0.08 Hz) performed on the time series of each voxel to remove the effects of low‐frequency drift and high‐frequency noise.

#### 2.4.3 Degree Centrality

We applied the so‐called degree centrality mapping analysis, which generates a degree centrality (DC) value for every voxel in the brain. The higher this value, the greater the connectedness of the respective brain area with the rest of the brain and thus the importance on the whole brain network. Specifically, a voxel‐based correlation analysis of the whole brain was performed on the preprocessed fMRI data, and Pearson's correlations were calculated for the time course from each voxel to every other voxel in the whole brain to create a correlation matrix. Then, the binarized matrix was obtained by thresholding each correlation at *r* > 0.25. These voxel‐wise DC values were converted into a *z*‐score matrix to improve normality and finally smoothed (FWHM = 8 mm) for statistical analysis. The researcher analysing all MRI data (SK) was blinded to the participant assignment.

### 2.5 Statistical Analysis

#### 2.5.1 General Data Analysis

As this was a first‐in‐man exploratory study and no prior human data existed to estimate effect sizes for glucagon‐related brain responses, formal a priori power calculations for region‐specific imaging outcomes were not feasible. In addition, the endogenous non‐suppressed glucagon group represented a naturally occurring phenotype within the cohort and was included primarily as a physiological reference group rather than as a separately powered discovery sample. Study conditions were applied as intention‐to‐treat. Analyses were performed in R (version 4.2.0). Due to the complex study design with repeated measures for some participants, we utilised generalized mixed effects models (*lme4* library) using the participant ID as a random effect. For serial OGTT trajectories, time was modelled using natural splines with 2 degrees of freedom only for analytes with repeated measurements across the full OGTT. For glucagon and NEFA, this corresponded to 7 timepoints (0, 15, 30, 60, 90, 120 and 150 min), allowing a parsimonious nonlinear trajectory fit. To assess whether nonlinear time modelling was warranted for the serial OGTT trajectories, we compared mixed‐effects models with linear time terms versus natural splines with 2 degrees of freedom using maximum‐likelihood estimation. For glucagon and NEFA, the spline models showed better fit than the linear models (glucagon: Akaike Information Criterion (AIC) 2235 vs. 2260, Bayesian Information Criterion (BIC) 2290 vs. 2304, χ^2^ = 31.4, *p* = 2.5 × 10^−6^; NEFA: AIC 3693 vs. 3744, BIC 3749 vs. 3788, χ^2^ = 56.4, *p* = 1.6 × 10^−11^), supporting the use of a parsimonious nonlinear time specification. Associations between changes in circulating glucagon and brain responses were assessed using repeated‐measures correlation (*rmcorr*). Effect sizes are reported as repeated‐measures correlation coefficients (r).

#### 2.5.2 Imaging Data Analysis

The DC and CBF maps were baseline adjusted (fMRI 30 min minus fMRI baseline and fMRI 120 min minus fMRI baseline). We performed whole‐brain paired *t*‐tests in SPM12 between saline and glucagon conditions for the baseline adjusted 30 min response and 120 min response separately (∆fMRI 30 min saline versus glucagon; ∆fMRI 120 min saline versus glucagon). A primary statistical threshold of *p* < 0.001 whole‐brain uncorrected and a *p* < 0.05 family wise error (FWE) corrected for multiple comparisons at the cluster level was applied. Small volume correction (SVC) was additionally applied for three a priori implicated in the hormonal regulation of food intake (hypothalamus, ventral tegmental area and ventral striatum) [29]. The masks were based on the AAL atlas 3 [30] and wfu_PickAtlas (https://www.nitrc.org/projects/wfu_pickatlas/). To correct for number of regions analysed, the SVC threshold was Bonferroni‐adjusted to p_FWE_ < 0.016. Brain regions showing significant changes in postprandial responsivity after glucagon compared to saline solution were extracted for post hoc analyses. The brain imaging outcomes were analysed at the prespecified postprandial timepoints of 30 and 120 min and were not modelled using splines. Because the primary whole‐brain analyses were based on within‐subject paired contrasts, time‐invariant participant characteristics such as age, sex, and educational attainment were inherently controlled for and were therefore not entered as additional voxel‐wise covariates. In the post hoc mixed‐effects analyses of extracted clusters, sex, age, and BMI were included as covariates. Educational attainment was not modelled separately because the cohort was relatively homogeneous in this respect, consisting predominantly of university students or academic staff with little socioeconomic variation. The paired whole‐brain saline‐versus‐glucagon analyses were performed in the intervention subgroup with paired resting‐state imaging under both conditions. These within‐subject analyses are distinct from the endogenous non‐suppressed reference group.

## 3 Results

After an overnight fast, all participants underwent a 75 g OGTT combined with infusion of either saline or glucagon in a single‐blind fashion (participants were told that the infusion could contain either saline or glucagon). Participants with previously documented glucagon suppression based on an earlier OGTT (i.e., lower glucagon concentrations at 120 min post OGTT compared to baseline) received glucagon infusion first, others with unknown status of glucagon suppression received a saline infusion first. The low‐dose glucagon (0.5 pmol/kg/min) or saline infusion started with the ingestion of 75 g glucose and lasted for 150 min.

Participants with unknown suppressed glucagon who initially received saline or with known glucagon suppression who received glucagon were invited to participate in a second OGTT. This OGTT was performed using the complementary setup (i.e., saline after glucagon, or glucagon after saline), resulting in a crossover design in a subset of participants (Figure 1). Functional MRI (fMRI) measurements were performed on each study day before the OGTT, after 30 min, and after 120 min. Blood was drawn every 30 min.

### 3.1 Glucagon Kinetics During OGTT and Peripheral Metabolism

Of the 30 participants who underwent OGTT, 22 had a suppression of their endogenous glucagon, while in 7, there was no such suppression. While fasting glucagon was not different between these groups (*p* = 0.8), post‐load glucagon was lower in those with suppressed glucagon (2 h‐post‐load glucagon, *p* < 0.001, Table 2). These groups did not differ in glycemia, insulin sensitivity or insulin secretion (all *p* > 0.1, Table 2).

**TABLE 2:** Anthropometric and metabolic characteristics of the study conditions shown as observed mean values (SD).

	non‐suppressed glucagon (I)	suppressed glucagon (II)	infusion‐induced glucagon rise (III)	*p*I vs II	*p*II vs III
*N*	7	22	14		
Male (*n* (%))	3 (42.9)	13 (59.1)	6 (42.9)	> 0.99	> 0.99
Age (years)	40 (17)	44 (14)	47 (13)	0.4	0.1
BMI (kg/m2)	25.4 (4.5)	25.7 (4.6)	24.8 (4.6)	0.8	0.9
HbA1c (%)	5.36 (0.34)	5.50 (0.41)	5.48 (0.27)	0.4	0.1
Fasting glucose(mmol/l)	5.1 (0.4)	5.1 (0.6)	5.0 (0.4)	0.4	0.4
Glucose at 120 min during OGTT (mmol/l)	5.4 (1.1)	5.5 (1.9)	5.6 (1.2)	0.7	0.2
AUC(0–120) glucose	831 (169)	835 (197)	796 (165)	0.7	> 0.99
Fasting glucagon (pg/dl)	65.1 (29.1)	74.9 (46.0)	69.1 (39.6)	0.8	0.5
Glucagon at 30 min during OGTT(pg/dl)	69.7 (33.9)	60.4 (25.1)	131.3 (54.1)	0.005	< 0.001
Glucagon at 120 min during OGTT (pg/dl)	72.9 (33.5)	59.1 (30.1)	169.1 (56.9)	< 0.001	< 0.001
AUC(0–120) glucagon	8372 (3950)	7773 (3934)	17 250 (5935)	< 0.001	< 0.001
Delta glucagon 0–120 (pg/dl)	7.8 (6.4)	−15.8 (19.5)	100.0 (58.5)	< 0.001	< 0.001
Insulin sensitivity, OGTT‐derived	16.5 (4.0)	14.4 (8.1)	12.8 (6.4)	0.1	0.3
Insulin secretion	288 (95)	273 (87)	321 (82)	0.4	0.3
NEFA suppression (dAUC)	19761.4 (5320.4)	27498.6 (12989.9)	27930.0 (12091.3)	0.05	0.9

*Note*: For clinical readability, raw descriptive values are presented here, while model‐estimated marginal means with 95% confidence intervals from the mixed‐effects models are provided in Table S5.

### 3.2 Effect of Low‐Dose Glucagon Infusion on Peripheral Metabolism

**FIGURE 2:**
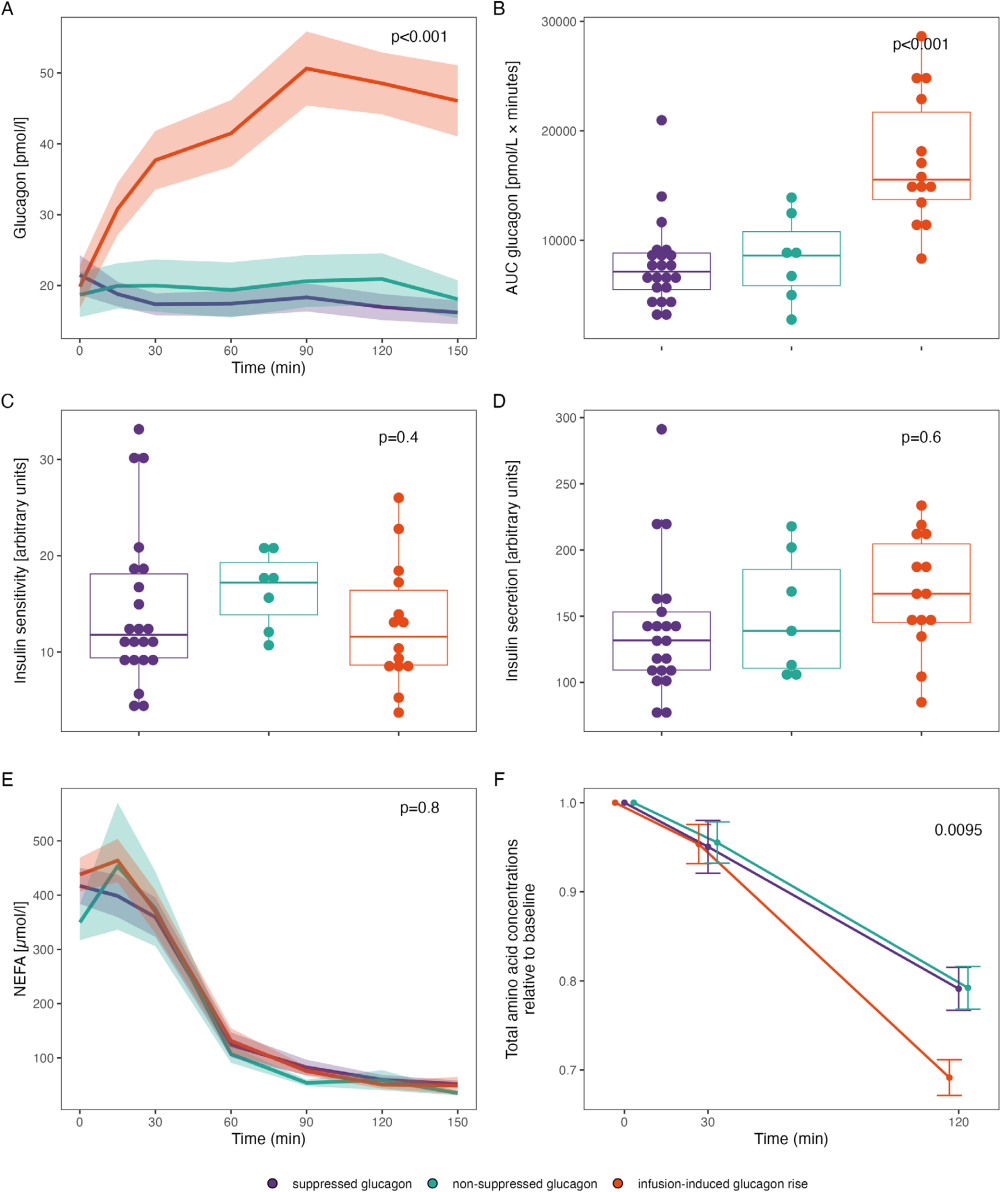
Change of plasma glucagon levels (A) and integrated plasma glucagon levels (B) during OGTT, stratified for the study condition. Panels C and D show insulin sensitivity and insulin secretion calculated from glucose and insulin levels. Panels E and F show NEFA and total amino acid levels respectively. Ribbons indicate standard errors (SE). For panel F, cumulated total amino acids (21 measured amino acids) relative to fasting are shown and SE was calculated only for the time‐points 30 and 120 (error bars, points slightly dodged to aid visibility). Statistical comparison was performed with generalized mixed effects models. For glucagon and NEFA (Panel A, E), time was modelled with natural splines (2 degrees of freedom), and its interaction with the condition was tested. In this analysis, time was employed in a linear fashion. For AUC glucagon, insulin sensitivity and insulin secretion, the *p* value for the condition is given. All models were adjusted for age, age‐squared (age^2^), BMI (log‐transformed) and sex.

Of the 22 participants with suppressed glucagon, 14 underwent another OGTT with glucagon infusion. While circulating glucagon was higher during infusion (AUC glucagon, *p* < 0.001, Table 2, Figure 2A,B), the low dose of glucagon infused did neither elevate fasting glucose nor the glucose levels during OGTT (all *p* ≥ 0.2, Table 2). Also, no changes in insulin sensitivity or insulin secretion were observed (both *p* = 0.3, Table 2, Figure 2C,D). In line, the suppression of non‐esterified fatty acids during OGTT, as a proxy for adipose tissue insulin action, was comparable between conditions (*p* = 0.05, Figure 2E). However, there were profound effects on circulating amino acids with a stronger suppression upon glucagon infusion (*p* = 0.0028, Figure 2F, Figure S1). While GLP‐1 levels were not different across conditions, there were differences in GIP levels consistent with a glucagon‐induced suppression (*p* = 0.02, Figure S2A,B).

### 3.3 Effect of Low‐Dose Glucagon Infusion on Postprandial Brain Responsivity

We examined resting‐state brain activity during OGTT under saline and glucagon infusion, analysing changes in cerebral blood flow and degree centrality, an indicator of whole‐brain functional connectivity, at 30 and 120 min after glucose ingestion, with adjustments for baseline levels.

**FIGURE 3:**
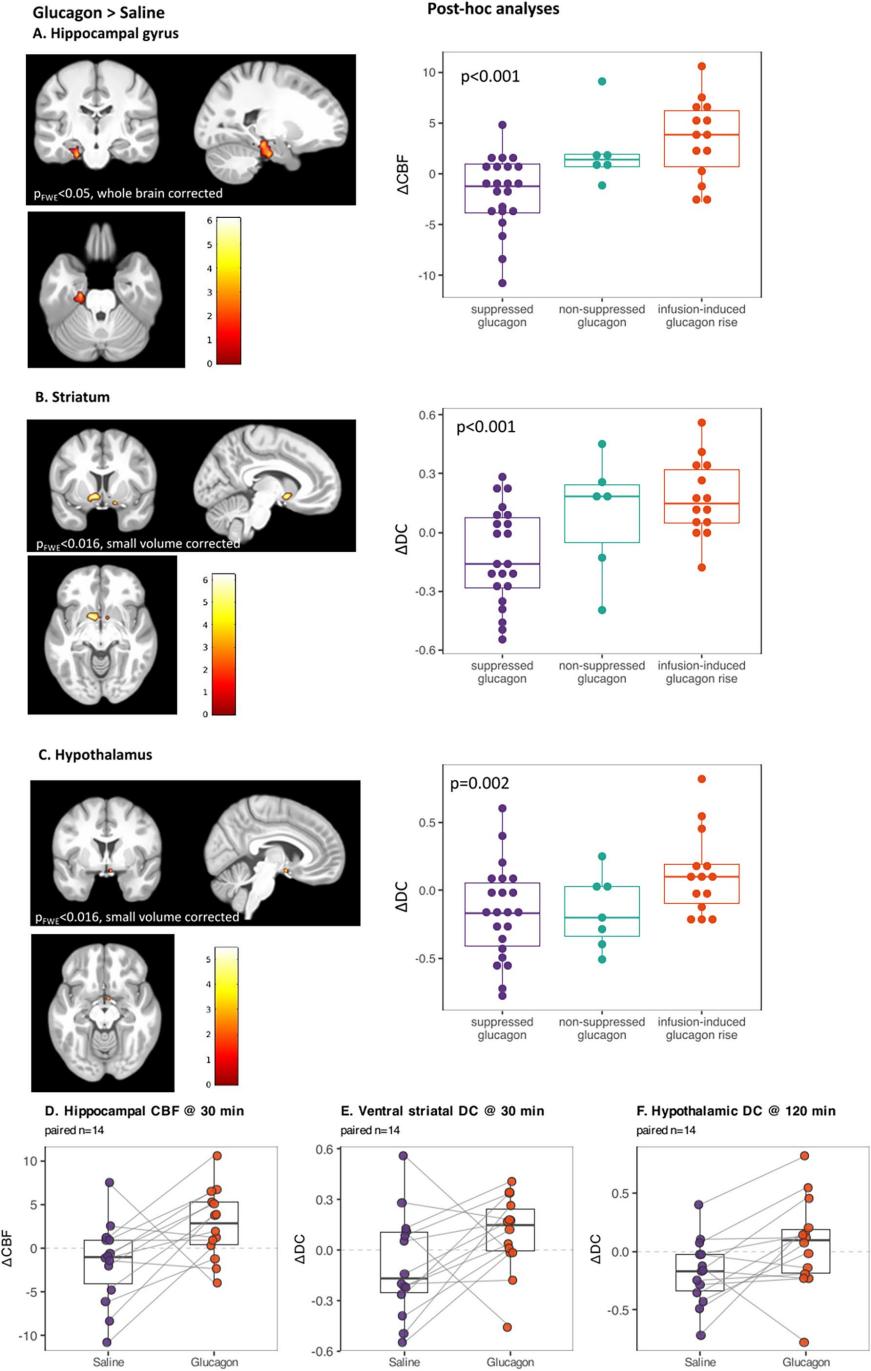
Postprandial changes in regional cerebral blood flow and functional connectivity during oral glucose tolerance test, stratified to glucagon condition. Colour‐coded *t*‐value maps represent significant voxels, *p* < 0.001 uncorrected for display. The accompanying extracted regional values are shown to illustrate the direction and distribution of the observed effects across conditions, including individual data points, and should be interpreted as descriptive visualisations. The primary inferential evidence for glucagon‐responsive regions is based on the corrected voxel‐wise analyses described in the Results and Methods. (A) Panel shows changes in cerebral blood flow (ΔCBF) in the hippocampal gyrus from before to 30 min after glucose ingestion. (B) Panel shows changes in resting‐state functional connectivity (change of degree centrality, ΔDC) in the striatum from before to 30 min after glucose ingestion. (C) Panel shows ΔDC in the hypothalamus from before to 120 min after glucose ingestion. Differences were assessed using generalized mixed effects models, with sex, age, and BMI included as fixed effects, and participants as a random effect. An ANOVA‐like test was applied to determine the significance of the fixed effect of the condition on the change in the respective variable, resulting in the reported *p* values. (D‐F) Paired within‐subject visualisation of the 14 participants who underwent both saline and glucagon infusion. Individual trajectories are shown together with boxplots for hippocampal CBF at 30 min (D), ventral striatal DC at 30 min (E), and hypothalamic DC at 120 min (F). The paired panels are intended as descriptive visualisation of the individual response patterns; primary inference was based on the corrected voxel‐wise analyses described in the Results and Methods.

First, we compared measurements during OGTT between saline and glucagon infusion conditions overall participants pairwise. The whole‐brain analysis revealed significant differences in the left hippocampal gyrus 30 min post glucose load with higher CBF after glucagon compared to saline (Figure 3A; peak voxel MNI coordinate x: –21, y: –28, z: –16; T (12) = 5.1; p_FWE_ < 0.05 corrected for multiple comparisons whole brain cluster level). No significant differences were observed 120 min post load (Table S3).

For post hoc analysis, we extracted CBF of the hippocampal gyrus, which revealed significant differences across the three conditions (*p* < 0.001, adjusted for sex, age, BMI; Figure 3A). At 30 min post glucose load, participants with suppressed glucagon had lower CBF than those with non‐suppressed glucagon. In those with initially suppressed glucagon, low‐dose glucagon infusion increased CBF, and direct post hoc comparison did not show a significant difference between the infusion‐induced glucagon rise group and the endogenous non‐suppressed glucagon group (*p* = 0.385; Table S2). However, the corresponding 95% confidence intervals were wide and did not establish formal equivalence (hippocampal CBF: estimate −2.34, 95% CI: 6.62–1.94; ventral striatum: estimate −0.086, 95% CI: 0.384–0.213; hypothalamus: estimate −0.322, 95% CI: 0.704–0.060; Table S2).

For the whole‐brain functional connectivity analysis, paired tests showed different degree centrality in the glucagon compared to saline condition 30 min post glucose load bilaterally in the ventral striatum (peak voxel MNI coordinates x: –10, y:8, z: –6; T (12)= 6.24; p_FWE_ < 0.016 small volume corrected) and 120 min post glucose load in the right hypothalamus (peak voxel MNI coordinate x: 6, y: 0, z: –12; T (12) = 4.10; p_FWE_ < 0.016 small volume corrected).

Post hoc analyses demonstrated significant differences in functional connectivity across the three groups/conditions (both *p* ≤ 0.002, adjusted for sex, age, BMI). At 30 min post glucose load, participants with suppressed glucagon had lower change in degree centrality (ΔDC) in the ventral striatum than those with non‐suppressed glucagon. This was increased by low‐dose glucagon infusion, with no significant difference between the infusion‐induced glucagon rise group and the endogenous non‐suppressed glucagon group (Figure 3B, *p* = 0.763; Table S2). In the hypothalamus, participants with non‐suppressed glucagon and those with suppressed glucagon were comparable. However, glucagon infusion increased the degree centrality (Figure 3C). The direct comparison between the infusion‐induced glucagon rise group and the endogenous non‐suppressed glucagon group at 120 min was not statistically significant (*p* = 0.112; Table S2).

The corresponding paired‐samples effect sizes were large (Cohen's d_z_ = 1.41 for hippocampal CBF at 30 min, 1.73 for ventral striatal degree centrality at 30 min, and 1.14 for hypothalamic degree centrality at 120 min). To illustrate the within‐subject response pattern underlying these paired analyses, individual saline‐versus‐glucagon trajectories for hippocampal CBF, ventral striatal DC, and hypothalamic DC are shown in Figure 3D–F.

In sensitivity analyses, adjustment for the first three amino‐acid principal components, explaining 88.9% of the variance in circulating amino acids, did not abolish the main group effects (left hippocampal CBF at 30 min: *p* = 0.00023; ventral striatal connectivity at 30 min: *p* = 0.00011; hypothalamic connectivity at 120 min: *p* = 0.0298). After additional adjustment for time‐matched GIP, the 30 min effects remained significant (left hippocampal CBF: *p* = 0.0022; ventral striatal connectivity: *p* = 0.0052), whereas the 120 min hypothalamic effect was attenuated (*p* = 0.191).

For all these brain areas, the changes in brain activity and functional connectivity were associated with the corresponding change in circulating glucagon, as assessed by repeated‐measures correlation, regardless of whether the glucagon was of exogenous or endogenous origin (Table S4). We did neither detect significant associations with changes in plasma glucose nor serum insulin (all *p* > 0.05), except for the hypothalamus, which was correlated to changes in serum insulin (*p* = 0.04).

The paired intervention subgroup was metabolically similar to the overall cohort (Table S6). In addition, leave‐one‐out analyses of the extracted degree‐centrality peaks showed that the ventral striatal and hypothalamic effects remained significant after omission of any single participant (Table S7), arguing against disproportionate influence by one individual.

## 4 Discussion

In this study, we investigated the effects of rising glucagon concentrations during an oral glucose tolerance test, a phenomenon previously observed to be more common in persons with a metabolically healthy, insulin sensitive phenotype [6]. A low‐dose glucagon infusion was designed as a physiological mechanistic probe to mimic non‐suppressed post‐challenge glucagon, rather than to elicit a pharmacological hyperglycemic response. As expected, the low‐dose glucagon infusion did not immediately affect insulin secretion, sensitivity, or post‐load glycemia, but induced the anticipated glucagon‐related changes in circulating amino acid levels [31, 32], consistent with the intended physiological rather than pharmacological intervention. At the same time, the chosen dose was sufficient to introduce robust glucagon signals to test brain effects experimentally. As a result, the achieved glucagon rise was somewhat higher than the average endogenous increase in the non‐suppressed group, but remained within the high end of the physiological range.

Our glucagon infusion protocol reproduced the early hippocampal and ventral striatal response pattern observed in endogenous glucagon non‐suppression, whereas the hypothalamic finding was less concordant. In the hypothalamus, increased degree centrality was observed after infusion but not clearly in the endogenous non‐suppressed group, which may reflect the somewhat higher glucagon rise achieved through infusion, a threshold‐like sensitivity of later hypothalamic responses, or additional indirect co‐signalling. We therefore interpret the infusion as partially recapitulating, rather than fully matching, the endogenous non‐suppressed phenotype. The comparisons between groups should therefore be interpreted as exploratory and hypothesis‐generating rather than as evidence of equivalence between these conditions.

The postprandial brain responses correlated with the rise in glucagon, regardless of whether the glucagon was of exogenous or endogenous origin. Adjustment for amino‐acid principal components did not abolish the main effects, arguing against a sole contribution of amino‐acid changes. However, attenuation of the 120 min hypothalamic effect after additional GIP adjustment indicates that indirect co‐signalling may contribute, particularly to later responses.

Overall, glucagon modulated brain activity in regions relevant for body weight [33], metabolic regulation, and cognition [15]. The best studied connection between the brain and glucagon is the stimulation of glucagon release by the brain in case of hypoglycemia [34]. Our current findings demonstrate that this connection is likely bi‐directional, that is, the brain is not only affecting pancreatic glucagon secretion but glucagon is also affecting the brain.

We detected changes in regional brain activity in response to glucagon infusion in three brain areas: The hypothalamus is a critical central regulator of whole‐body metabolism [35]. Besides numerous further homeostatic functions, it has the ability to modulate endogenous glucose production [36, 37] and regulate the secretion of pancreatic hormones [38, 39, 40]. Of note, recent research indicates that this function might not be limited to rodents but appears to also exist in humans, where this has been studied especially in response to brain insulin action [38, 41]. Furthermore, the hypothalamus is key in regulating food intake [42, 43]. Presumably through a concert of these functions, human hypothalamic activity is linked to an increase in body weight and visceral adipose tissue [17, 43]. Glucagon appears to contribute to these hypothalamic pathways: central glucagon injections in animals reduced food intake through glucagon's action in the hypothalamus [13, 44, 45]. In rodent experiments, hypothalamic glucagon action was furthermore able to modulate systemic glycemia [44, 46].

In rodents, glucagon action in the brainstem, particularly in the dorsal vagal complex (DVC), has also been described [14]. This region is key in regulating glucose and energy balance through the autonomic nervous system [14]. In our current study, we did not detect significant glucagon effects in the brainstem. This discrepancy may be due to methodological limitations in neuroimaging, as brainstem nuclei are small and prone to susceptibility artefacts in fMRI [47], which may reduce sensitivity to detect subtle changes in this region. In addition, differences between species in the relative contribution or accessibility of the brainstem for glucagon actions cannot be excluded.

Our study revealed that glucagon's influence extends beyond the hypothalamus, affecting the hippocampus, a key area for memory formation [48] and known to express glucagon receptors [11]. Recent evidence demonstrates glucagon receptor expression in human brain tissue beyond the blood–brain barrier, supporting the plausibility of direct central glucagon action [12]. We also observed glucagon's central effects in the striatum, which plays a key role in motor control and reward processing [49], and is the main projection site of dopamine neurons. This link merits further investigation, especially considering reported potential neurocognitive benefits of GLP1‐analogues, which include improved hippocampal MRI biomarkers in patients with and without type 2 diabetes [50]. Additionally, both the hippocampus and striatum have been recently recognised for their roles in whole‐body metabolism and appetite regulation [15], suggesting that glucagon's action in these areas could enhance metabolic control.

Differences between the groups during OGTT were most evident in the hippocampus and ventral striatum. In participants with suppressed glucagon, low‐dose glucagon infusion increased these responses toward the pattern observed in those with endogenous non‐suppressed glucagon, consistent with physiological variation in post‐challenge glucagon contributing to interindividual differences in central postprandial responsivity.

Together with our previous findings linking non‐suppressed glucagon to a more favourable metabolic phenotype [6], the present data are consistent with a role of the brain as one component of this phenotype. However, the current study was not designed to establish the precise mechanisms mediating these effects, and possible contributions of receptor saturation, indirect hormonal co‐signalling, or other downstream pathways should therefore be addressed in larger mechanistic studies. In addition, the modest sample size and the partly observational nature of the comparisons limit statistical power and preclude definitive conclusions regarding functional significance.

Glucagon's action in these brain areas could be of clinical importance, as glucagon's ability to reduce body weight depends on the brain [1]. Hence, differences in glucagon action in the human brain could contribute to the differences in body weight and whole‐body metabolism between persons with a suppressed versus non‐suppressed glucagon during an OGTT.

An important aspect to consider is the potential for glucagon resistance in the human brain, just as for other metabolic hormones such as insulin [16] and leptin [51], for example, in obesity. Research in rodents and data on glucagon effects on peripheral metabolism in humans [31] suggests the possibility of such a brain glucagon resistance. It will be interesting to further explore this in humans and clarify potential metabolic consequences.

What sets our study apart from others is its focus on glucagon's role during an OGTT, specifically capturing postprandial effects. After food intake, a plethora of hormones and metabolites exert effects on the brain [52]. Glucagon likely acts in concert with incretin hormones and insulin, signalling to the brain about current food intake and triggering brain‐derived responses to manage energy availability.

Interestingly, recent pharmacological approaches for the treatment of diabetes and obesity address such co‐signalling pathways by simultaneously targeting peptide hormone receptors. Multi‐agonists that also address the glucagon receptor achieved marked effects on body weight, food intake [53] and metabolism [54]. Our findings support the concept that glucagon may recruit brain circuits that partly overlap with those engaged by incretin hormones, providing a potential CNS mechanism through which co‐agonism could enhance metabolic and behavioural effects. In translational studies, glucagon‐related brain responsivity during a metabolic challenge may serve as a mechanistic readout to assess brain glucagon action in humans. Future studies should determine whether GLP‐1/glucagon or GLP‐1/GIP/glucagon co‐agonists alter hypothalamic, striatal and hippocampal activity or connectivity, and whether these brain effects translate into changes in food intake, body weight, or whole‐body metabolism.

Although the mixed‐effects modelling increased statistical efficiency, the modest sample size, especially in subgroup analyses, limited power to detect smaller effects; therefore, region‐specific findings should be interpreted cautiously and require replication in larger studies. Further limitations of our study include the fact that the achieved glucagon concentrations through infusion were at the high end of the physiological range and therefore do not reflect typical postprandial glucagon concentrations in most persons, although they remained within a physiological range observed in some individuals. In addition, brain responses were assessed only within the first 120 min after glucose ingestion, and thus the persistence of brain effects beyond the acute postprandial period remains unclear. In addition, participation in the crossover component was based on availability and willingness to return for a second visit, which may introduce selection bias. Moreover, no behavioural or energy expenditure endpoints were assessed, limiting conclusions regarding downstream functional consequences for body weight regulation.

## 5 Conclusion

Our study provides insights into glucagon action beyond traditional metabolic pathways by demonstrating effects of high physiological glucagon concentrations on the human brain. Experimentally elevating glucagon during an OGTT increased brain responsivity in areas important for homeostatic and hedonic regulation of eating behaviour, whole‐body metabolism, and cognition. Thus, brain glucagon signalling could contribute to the observed metabolic and weight differences between persons with non‐suppressed versus suppressed glucagon during an OGTT [6], positioning the brain as one important organ of glucagon action. These effects may also underlie the promising effects on body weight achieved with upcoming pharmacological multi‐agonists that activate the glucagon receptor [54].

## Author Contributions

R.W. and M.H. designed the study, performed experiments, analysed data and wrote the manuscript. S.K. performed experiments, analysed data and wrote the manuscript. J.H. researched data, contributed to manuscript drafting and contributed to discussion. K.P. supported statistical analyses and contributed to discussion. E.H. performed experiments. R.V., A.L.B. and H.P. contributed to discussion of results. H.‐U.H. and A.F. contributed to study design and to discussion. A.P. supervised laboratory measurements and contributed to discussion. All authors approved the final version of the manuscript before submission.

## Funding

The study was supported in parts by a grant (01GI0925) from the Federal Ministry of Education and Research (BMBF) to the German Center for Diabetes Research (DZD). The BMBF did not influence the design, conduct, analysis and reporting of the trial.

## Disclosure

R.W. and S.K. are the guarantors of this work and, as such, had full access to all the data in the study and takes responsibility for the integrity of the data and the accuracy of the data analysis.

## Conflicts of Interest

Outside of the current work, R.W. reports lecture fees from Novo Nordisk, Sanofi‐Aventis, Boehringer‐Ingelheim, and Eli Lilly. He served on the advisory board for Akcea Therapeutics, Daichii Sankyo, Sanofi‐Aventis, Eli Lilly and NovoNordisk. Outside of the current work, M.H. reports research grants from Boehringer Ingelheim and Sanofi (both to the University Hospital of Tübingen) and lecture fees from Amryt, AstraZeneca, Bayer, Boehringer Ingelheim, Lilly, Novartis, Novo Nordisk and Sanofi. He also served on advisory boards for Boehringer Ingelheim, Sanofi and Amryt. None of the other authors report a conflicts of interest.

## Supporting information


**Table S1:** Sampling timepoints and the measures obtained at each timepoint during the OGTT.
**Table S2:** Extracted brain outcomes, adjusting for sex, age, and BMI and applying Tukey correction for multiple comparisons in the mixed‐model framework.
**Table S3:** Effect of glucagon versus saline infusion during the oral glucose tolerance test in the whole‐brain analysis. Whole brain data were analysed using paired‐*t*‐tests in SPM12 (glucagon vs. saline) using baseline adjusted cerebral blood flow (ΔCBF) and degree centrality (ΔDC) images for time point 30 min and 120 min adjusted for baseline separately. No significant differences were found for ΔCBF at time point 120 min. No significant differences were observed for saline minus glucagon. *p < 0.05, family wise error corrected for multiple comparisons, whole‐brain cluster level; †*p* < 0.016, small volume corrected.
**Table S4:** Repeated‐measures correlations between changes in circulating glucagon and brain responses.
**Table S5:** Model‐estimated marginal means with 95% confidence intervals from the mixed effects models. Conditions pairwise compared using generalized mixed regression models with participant as random effect and condition as fixed effect.
**Table S6:** Baseline characteristics of the overall cohort and the paired intervention subgroup. Data are presented as mean (SD) unless otherwise indicated.
**Table S7:** Leave‐one‐out sensitivity analysis on the extracted degree‐centrality peak values for the paired intervention subgroup.
**Figure S1:** Amino acid levels relative to baseline (fasting) during OGTT, measured at 3 timepoints, stratified for the 3 conditions (see colour legend). Comparisons were performed by linear mixed regression, and *p* values are given for the time over condition (time * condition interaction). Models were using additionally adjusted for age, age2, BMI (log transformed) and sex.
**Figure S2:** Mean GLP‐1 (A, *N* = 27) and GIP (B, *N* = 27) levels measured at 3 time‐points during OGTT, stratified for the study condition (see colour legend). Ribbons indicate standard errors. Courses of GLP‐1 and GIP were compared by generalized mixed regression. Time was modelled with natural splines (2 degrees of freedom) by analysing its interaction with the condition. The models were additionally adjusted for age, age2, BMI (log‐transformed) and sex.

## Data Availability

The data are not publicly available due to them containing information that could compromise research participant privacy/consent.
