# Supplementary material for: Postprandial Glucagon Action in the Human Brain
Source: Diabetes Obes Metab. 2026 Apr 22;28(7):5926–38. doi: 10.1111/dom.70801 (PMC13243955; doi:10.1111/dom.70801)

Supplementary Tables and Figures

Supplementary Table 1

Sampling timepoints and the measures obtained at each timepoint during the OGTT

Time after glucose ingestion	Measures obtained
0 min (fasting)	Plasma glucose, serum insulin, serum C-peptide, plasma glucagon, plasma NEFA; additionally total GLP-1, total GIP, and amino-acid profiling
15 min	Plasma glucose, serum insulin, serum C-peptide, plasma glucagon, plasma NEFA
30 min	Plasma glucose, serum insulin, serum C-peptide, plasma glucagon, plasma NEFA; additionally total GLP-1, total GIP, and amino-acid profiling; fMRI acquisition
60 min	Plasma glucose, serum insulin, serum C-peptide, plasma glucagon, plasma NEFA
90 min	Plasma glucose, serum insulin, serum C-peptide, plasma glucagon, plasma NEFA
120 min	Plasma glucose, serum insulin, serum C-peptide, plasma glucagon, plasma NEFA; additionally total GLP-1, total GIP, and amino-acid profiling; fMRI acquisition
150 min	Plasma glucose, serum insulin, serum C-peptide, plasma glucagon, plasma NEFA

Supplementary Table 2

Extracted brain outcomes, adjusting for sex, age, and BMI and applying Tukey correction for multiple comparisons in the mixed-model framework.

**Post-hoc comparisons**		**(non-suppressed glucagon) - (infusion-induced glucagon rise)**	**suppressed glucagon - (infusion-induced glucagon rise)**	**suppressed glucagon - (non-suppressed glucagon)**
1	ΔCBF_Parahippo_30min	0.38	<0.001	0.1
2	ΔDC_Hypothalamus_120min	0.11	0.0028	1
3	ΔDC_ventral-striatum_30min	0.76	<0.001	0.077

Supplementary Table 3

Effect of glucagon versus saline infusion during the oral glucose tolerance test in the whole-brain analysis. Whole brain data were analyzed using paired-t tests in SPM12 (glucagon vs. saline) using baseline adjusted cerebral blood flow (ΔCBF) and degree centrality (ΔDC) images for time point 30 min and 120 min adjusted for baseline separately. No significant differences were found for ΔCBF at time point 120 min. No significant differences were observed for saline minus glucagon. *p<0.05, family wise error corrected for multiple comparisons, whole-brain cluster level; †p<0.016, small volume corrected.

Peak Voxel (Name of Region)	Hem	MNI (mm)<br>(x,y,z)	T value
Glucagon minus Saline			
ΔCBF 30 min			
Hippocampal gyrus	Left	-21, -28, -16	5.09*
ΔCBF 120 min			
No differential activation			
ΔDC 30min			
Ventral Striatum	Left	-10, 8, -6	6.24†
ΔDC 120min			
Hypothalamus	Right	6, 0, -12	4.10†
Saline minus Glucagon			
No differential activation for ΔCBF and ΔDC 30 and 120 min			

Supplementary Table 4

Repeated-measures correlations between changes in circulating glucagon and brain responses

**Brain outcome**	**Timepoint**	**Repeated-measures correlation coefficient (r)**	**p value**
ΔCBF_Hippo	30 min	0.789	0.001
ΔDC_ventral-striatum	30 min	0.709	0.007
ΔDC_Hypothalamus	120 min	0.667	0.009

Supplementary Table 5

Model-estimated marginal means with 95% confidence intervals from the mixed-effects models. Conditions pairwise compared using generalized mixed regression models with participant as random effect and condition as fixed effect.

Measure	Non-suppressed glucagon, estimated marginal mean (95% CI)	Suppressed glucagon, estimated marginal mean (95% CI)	Infusion-induced glucagon rise, estimated marginal mean (95% CI)
age (years)	40 (29, 51)	45 (38, 51)	45 (39, 51)
BMI (kg/m2)	25.4 (21.8, 29.0)	26.0 (24.0, 28.0)	25.9 (23.9, 28.0)
HbA1c (%)	5.36 (5.07, 5.65)	5.50 (5.34, 5.66)	5.41 (5.24, 5.58)
glucose fasting (mmol/l)	5.1 (4.7, 5.5)	5.1 (4.9, 5.4)	5.1 (4.8, 5.3)
glucose 2h post-challenge (mmol/l)	5.4 (4.2, 6.7)	5.5 (4.8, 6.2)	5.8 (5.0, 6.6)
AUC(0-120) glucose	831 (684, 977)	826 (744, 907)	826 (736, 917)
glucagon fasting (pg/dl)	65.1 (33.8, 96.5)	75.4 (57.8, 93.0)	67.4 (46.1, 88.7)
glucagon 30 min (pg/dl)	69.6 (40.3, 99.0)	60.4 (43.4, 77.5)	131.3 (109.9, 152.8)
glucagon 120 min (pg/dl)	72.9 (41.4, 104.3)	58.9 (41.1, 76.7)	168.5 (145.7, 191.4)
delta glucagon 0-120 (pg/dl)	7.7 (-20.0, 35.5)	-16.0 (-31.7, -0.3)	99.8 (79.7, 120.0)
AUC(0-120) glucagon	8372 (4804, 11940)	7772 (5755, 9790)	17193 (14611, 19775)
insulin sensitivity, OGTT-derived	16.5 (11.1, 21.8)	14.3 (11.3, 17.3)	12.8 (9.4, 16.2)
insulin secretion	288 (216, 359)	279 (239, 318)	289 (248, 330)
NEFA suppression (dAUC)	19761 (10823, 28700)	27476 (22323, 32630)	27994 (21728, 34261)

Supplementary Table 6

Baseline characteristics of the overall cohort and the paired intervention subgroup. Data are presented as mean (SD) unless otherwise indicated.

**Characteristic**	**Overall cohort**	**Paired intervention subgroup**
n	30	12
female/male	14/16	6/6
age (years)	43.5 (14.7)	45.7 (14.4)
BMI (kg/m2)	25.9 (4.6)	24.8 (5.8)
fasting glucose (mmol/L)	5.2 (0.5)	5.1 (0.5)
glucose 120 min (mmol/L)	5.5 (1.7)	5.4 (1.6)
fasting glucagon (pg/dL)	76.6 (43.7)	80.9 (56.9)
Matsuda index	14.3 (7.5)	14.4 (8.6)

Supplementary Table 7

Leave-one-out sensitivity analysis on the extracted degree-centrality peak values for the paired intervention subgroup

Region	Time (min)	Mean difference range	p-value range
Hypothalamus	120	0.296 to 0.385	0.0011 to 0.0053
Ventral striatum	30	0.276 to 0.355	0.0003 to 0.0024

Supplementary Figure 1

Amino acid levels relative to baseline (fasting) during OGTT, measured at 3 timepoints, stratified for the 3 conditions (see color legend). Comparisons were performed by linear mixed regression, and p-values are given for the time over condition (time * condition interaction). Models were using additionally adjusted for age, age^2, BMI (log-transformed) and sex.

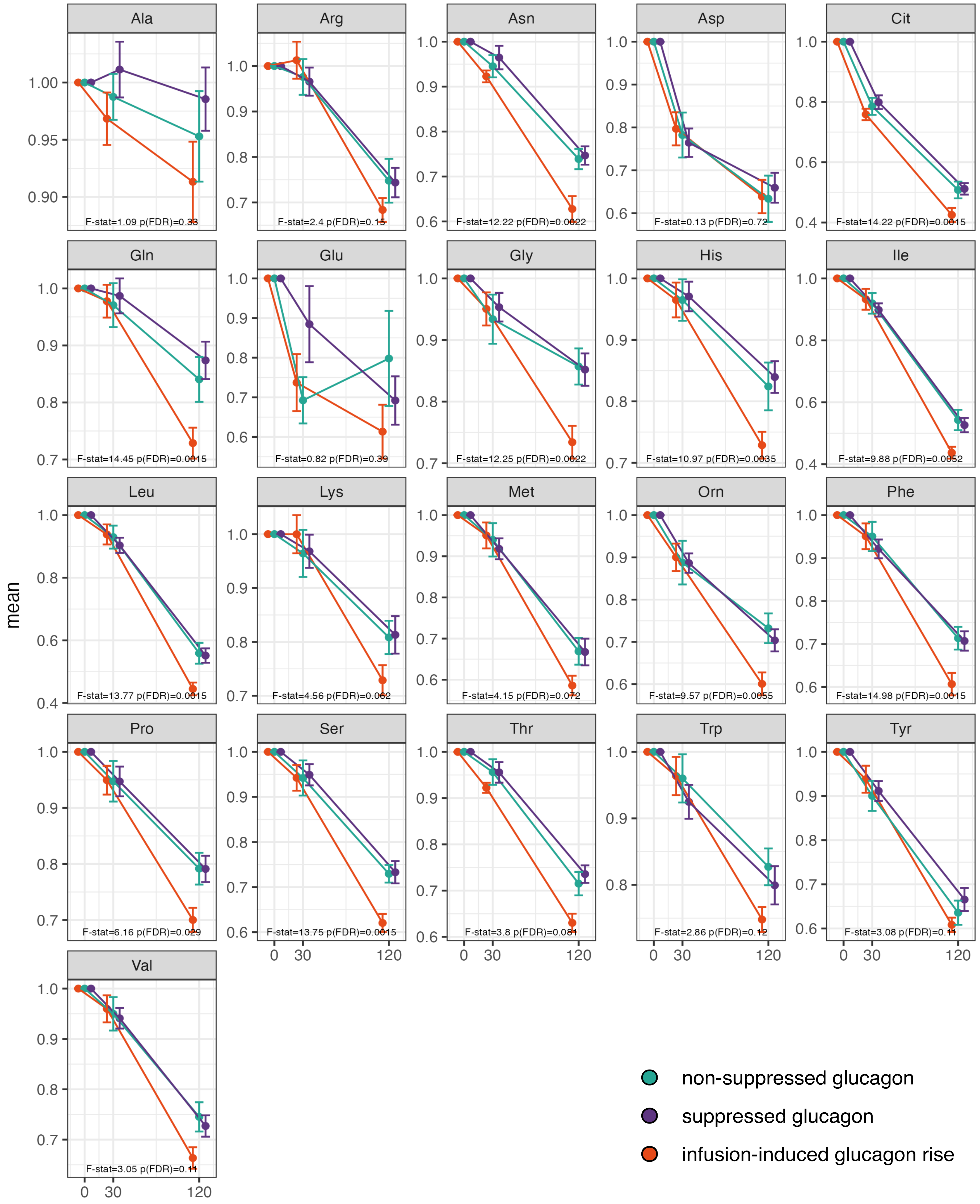

Supplementary Figure 2

Mean GLP-1 (A, N=27) and GIP (B, N=27) levels measured at 3 time-points during OGTT, stratified for the study condition (see color legend). Ribbons indicate standard errors. Courses of GLP-1 and GIP were compared by generalized mixed regression. Time was modelled with natural splines (2 degrees of freedom) by analyzing its interaction with the condition. The models were additionally adjusted for age, age^2, BMI (log-transformed) and sex.

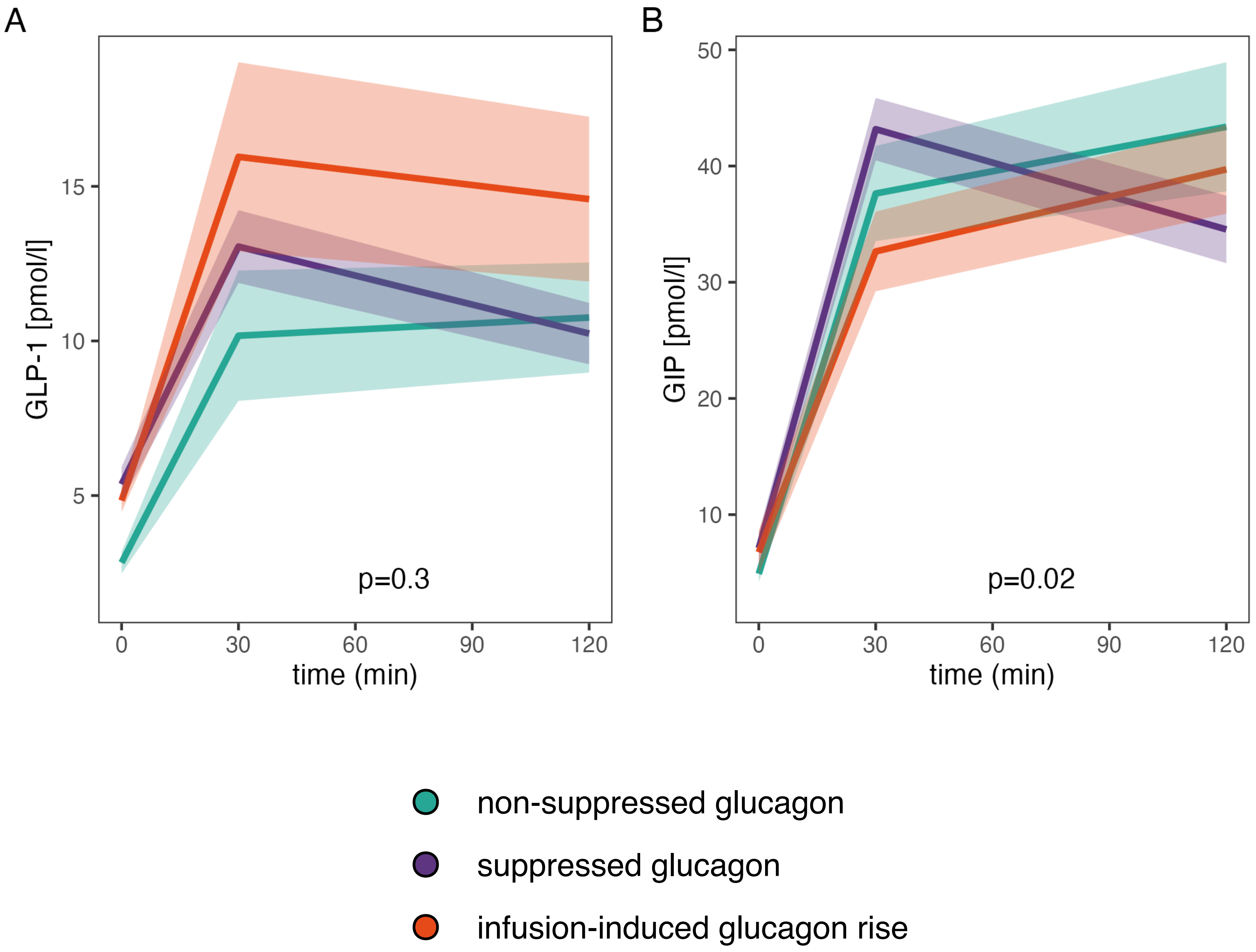

Supplement: Supplementary file 1 — Table S1: Sampling timepoints and the measures obtained at each timepoint during the OGTT. Table S2: Extracted brain outcomes, adjusting for sex, age, and BMI and applying Tukey correction for multiple comparisons in the mixed‐model framework. Table S3: Effect of glucagon versus saline infusion during the oral glucose tolerance test in the whole‐brain analysis. Whole brain data were analysed using paired‐t‐tests in SPM12 (glucagon vs. saline) using baseline adjusted cerebral blood flow (ΔCBF) and degree centrality (ΔDC) images for time point 30 min and 120 min adjusted for baseline separately. No significant differences were found for ΔCBF at time point 120 min. No significant differences were observed for saline minus glucagon. *p < 0.05, family wise error corrected for multiple comparisons, whole‐brain cluster level; †p < 0.016, small volume corrected. Table S4: Repeated‐measures correlations between changes in circulating glucagon and brain responses. Table S5: Model‐estimated marginal means with 95% confidence intervals from the mixed effects models. Conditions pairwise compared using generalized mixed regression models with participant as random effect and condition as fixed effect. Table S6: Baseline characteristics of the overall cohort and the paired intervention subgroup. Data are presented as mean (SD) unless otherwise indicated. Table S7: Leave‐one‐out sensitivity analysis on the extracted degree‐centrality peak values for the paired intervention subgroup. Figure S1: Amino acid levels relative to baseline (fasting) during OGTT, measured at 3 timepoints, stratified for the 3 conditions (see colour legend). Comparisons were performed by linear mixed regression, and p values are given for the time over condition (time * condition interaction). Models were using additionally adjusted for age, age2, BMI (log transformed) and sex. Figure S2: Mean GLP‐1 (A, N = 27) and GIP (B, N = 27) levels measured at 3 time‐points during OGTT, stratified for the study conditi [file DOM-28-5926-s001.pdf]
